# Of mice and lymphoid aggregates: modeling tertiary lymphoid structures in cancer

**DOI:** 10.3389/fimmu.2023.1275378

**Published:** 2023-10-26

**Authors:** Alessandra Vaccaro, Tiarne van de Walle, Mohanraj Ramachandran, Magnus Essand, Anna Dimberg

**Affiliations:** Department of Immunology, Genetics and Pathology, Science for Life Laboratory, The Rudbeck Laboratory, Uppsala University, Uppsala, Sweden

**Keywords:** tertiary lymphoid structures, murine models, cancer, immunotherapy, antigen-presenting niches

## Abstract

Tertiary lymphoid structures (TLS) are lymph node-like aggregates that can form in association with chronic inflammation or cancer. Mature TLS are organized into B and T cell zones, and are not encapsulated but include all cell types necessary for eliciting an adaptive immune response. TLS have been observed in various cancer types and are generally associated with a positive prognosis as well as increased sensitivity to cancer immunotherapy. However, a comprehensive understanding of the roles of TLS in eliciting anti-tumor immunity as well as the mechanisms involved in their formation and function is still lacking. Further studies in orthotopic, immunocompetent cancer models are necessary to evaluate the influence of TLS on cancer therapies, and to develop new treatments that promote their formation in cancer. Here, we review key insights obtained from functional murine studies, discuss appropriate models that can be used to study cancer-associated TLS, and suggest guidelines on how to identify TLS and distinguish them from other antigen-presenting niches.

## Introduction

Tertiary lymphoid structures (TLS) are ectopic immune aggregates that form at sites of chronic inflammation such as cancer (1). By definition, TLS are tight clusters of lymphoid cells that can organize in distinct B and T cell compartments, thus resembling secondary lymphoid organs. B cell-rich areas of these structures can contain evidence of B cell class switching, actively proliferating B cells in germinal centers (GCs) as well as follicular dendritic cells (FDCs). TLS also contain antigen-presenting cells (APCs) and can form in close proximity of high endothelial venules (HEVs) (2–5). TLS maturation has been categorized into three stages: (i) early TLS, characterized by dense lymphocytic aggregates without follicular dendritic cells (fDCs); (ii) primary follicle-like TLS, having B cell clusters and an fDC network without GCs; and (iii) secondary follicle-like TLS, which includes B cell clusters, an fDC network, and GCs (6, 7). However, recent advances indicate that the maturation of TLS is a dynamic process which can vary depending on the underlying chronic inflammatory condition and the specific tissue involved (6, 8, 9).

TLS have been identified in numerous cancer types, and their correlation with extended survival and response to immunotherapy has been extensively explored (10, 11), establishing TLS as robust biomarkers with potential clinical utility in patient stratification. Due to their resemblance to lymph nodes in terms of organization, it is speculated that TLS may serve as local sites for initiating anti-tumor immune responses in cancer. Nevertheless, the precise biological role and function of these structures remain to be elucidated. While association-based studies can be conducted using biobanked patient tissues, functional studies necessitate dynamic systems where different variables can be manipulated to address specific questions. Murine models of cancer, when appropriately employed to replicate the human scenario, offer powerful tools to shed light on these questions and obtain insights into the impact of TLS on tumor biology and immune responses. Here, we highlight important advances in the understanding of TLS biology, which were made possible by functional studies in mice, and discuss critical aspects to consider when modeling cancer-associated TLS.

## Unleashing the potential of orthotopic models to explore TLS in cancer

The significant variation in composition, maturity, and localization of TLS among different cancer types is becoming increasingly evident. This diversity can be attributed to the organ-specific microenvironment where tumors develop. Notable examples include gastrointestinal cancers, which are influenced by the intestinal flora, metastatic cancers that establish in a non-primary niche, and cancers of the central nervous system, where immune responses are tightly regulated and often suppressed. Unraveling cancer-specific functions of TLS necessitates the use of orthotopic, immunocompetent models that not only replicate the tumor phenotype but also faithfully mimic its anatomical location and interactions with the immune system.

Characteristics of the tumor itself as well as the local microenvironment can affect the immune response and formation of TLS. For instance, although TLS are observed in the vast majority of colorectal cancer (CRC) patients (6, 12), the level of their maturation varies (6). For instance, patients with microsatellite instability-high (MSI-H) and/or BRAF mutations exhibit a higher number of mature secondary follicle-like TLS, indicating that these genetic alterations can influence TLS maturation in CRC (6). Interestingly, investigations using the orthotopic azoxymethane-dextran sodium sulfate (AOM/DSS) murine model of CRC revealed that specific gut flora also play a role in determining TLS maturation (13). Indeed, colonization of Helicobacter hepaticus (Hhep) in the colon of CRC-bearing mice was associated with increased percentages of mature TLS, which contained Hhep-specific T follicular helper (Tfh) cells that proved crucial for TLS formation, immune infiltration, and tumor control (13). These findings provide insights into how microbiota-specific Tfh cells can influence the development of mature TLS in CRC.

The impact of the tumor’s location on the formation of TLS was elegantly demonstrated by Rodriguez et al. (14), who observed the presence of TLS in B16-OVA melanomas when the tumors were implanted intraperitoneally (i.p.), but not when they were grown subcutaneously (s.c.). The i.p. injected tumors were located below the stomach and above the cecum, in association with the spleen and omentum, simulating visceral metastasis rather than cutaneous melanoma (14). This finding is consistent with observations in patients, where TLS with distinct B and T cell zones are more commonly found in the metastatic setting (15). Interestingly, the authors revealed that a population of PDPN^hi^FAP^neg^ cancer-associated fibroblasts (CAFs), which was more abundant in i.p. tumors compared to s.c. tumors, acted as lymphoid tissue organizer cells and was responsible for TLS formation (14). The availability of such CAFs in the subcutaneous versus peritoneal environment could explain the lower incidence of zonated TLS in cutaneous versus metastatic melanoma in patients (15).

Another study highlighting the potential of murine models in understanding TLS function in cancer utilized immunocompetent xenograft models of CRC. Similar to melanoma, CRC is also a metastatic cancer and can form TLS in lung and liver metastases (16–18). In this study, the authors orthotopically implanted patient-derived organoids in NSG mice with a humanized immune system, creating a model of metastatic CRC with a primary mass and distal metastases in the liver and peritoneum (19). Employing this model, the authors were able to demonstrate that checkpoint blockade against PD-1 or CTLA-4 induced TLS formation in the primary tumor and liver metastases, but not in peritoneal metastases. Intriguingly, CRC patients with peritoneal metastases are usually resistant to immune checkpoint blockade (20). This work additionally showed that B cell depletion impaired the clearance of liver metastases post α-CTLA-4 therapy (19), emphasizing the functional role of B cells and TLS in anti-CRC immunity.

Furthermore, murine models played a crucial role in the identification of TLS in human glioma. While TLS had been previously reported in various peripheral cancers, their presence in glioma was not documented until 2021 (21, 22). In the syngeneic, orthotopic GL261 and CT-2A murine glioma models, van Hooren & Vaccaro et al. demonstrated TLS formation in proximity of the meninges rather than within the tumor mass (22). Routine core biopsies of glioma rarely contain meningeal tissue, which likely contributed to the oversight of TLS in this cancer type. The observation made in murine glioma was pivotal in defining the inclusion of meningeal tissue as a requirement in their patient cohort, leading to the identification of TLS in 8 out of 16 glioblastoma (GBM) samples (22). Similar to the murine setting, the authors found that TLS mostly formed in close proximity to meningeal tissues in human GBM. However, a smaller number of cases with intratumoral or peritumoral TLS were also observed (22). A subsequent study by Mauldin et al. detected intratumoral lymphoid aggregates resembling immature TLS in only 8% of the assessed GBM cases, however this study did not consider the availability of meningeal tissue as a parameter for selection, potentially overlooking meningeal TLS (23).

In conclusion, orthotopic murine models have demonstrated significant utility in advancing our understanding of TLS in cancer. These models effectively complement human tissue studies by enabling a comprehensive investigation into the tumor-specific attributes and functions of TLS.

## Unlocking the secrets of TLS induction: targeting the LTβR signaling axis

TLS are linked to positive prognosis in cancer, and as such their induction is an attractive therapeutic strategy for improving clinical outcomes. Several approaches to induce TLS have been explored in preclinical cancer models, with promising results. The lymphotoxin-β receptor (LTβR) signaling axis is crucial for the development of lymphoid tissues, making it a prime target in this respect. In murine cancer models, TLS formation has been successfully triggered using its two ligands, LTα_1_β_2_ and LIGHT/TNFSF14.

Engineering the murine glioma models GL261 and CT-2A to overexpress either LTα_1_β_2_ or LIGHT induced T cell-rich TLS, which was recapitulated when delivering LIGHT with an adeno-associated viral vector that specifically targets brain endothelial cells (AAV-LIGHT) (24). The presence of LIGHT in the tumor microenvironment (TME) also correlated with prolonged survival, increased presence of tumor-associated HEVs (TA-HEVs), enhanced effector/memory T cell responses, and formation of intratumoral antigen-presenting niches containing stem-like T cells. This data suggests that the composition of TLS can be therapeutically skewed to increase their T cell content and promote enhanced anti-tumor immunity. Increased TLS formation and prolonged survival post-AAV-LIGHT therapy were also observed in the mGC1 glioma model, which is expected to have a lower mutational burden compared to chemically-induced GBMs such as GL261 and CT-2A (24, 25). However, TLS occurrence in untreated mGC1-bearing mice was lower than in the chemically-induced models, suggesting that the mutational status of GBM tumors could influence TLS development.

Targeting LIGHT/TNFSF14 to tumor vessels through fusion to a vascular-targeting peptide (LIGHT-VTP) prolonged survival and sensitized RIP1-Tag5 pancreatic tumors to immunotherapies, while inducing formation of TLS and PNAd^+^ TA-HEVs (26). Interestingly, intraperitoneal injection of peritoneal macrophages pre-stimulated with LIGHT-VTP also induced both TLS and TA-HEVs while depletion of T cells resulted in their reduction, illustrating the important roles of macrophages and T cells in LIGHT-induced TLS formation. In line with this, treatment of orthotopically implanted KPC119 and Panc02 pancreatic ductal adenocarcinoma (PDAC) models with Nano-sapper, a tumor stroma-targeted calcium phosphate liposome carrying anti-fibrotic α-mangostin and a plasmid encoding LIGHT, enhanced TLS formation and T cell infiltration (27). Additionally, Nano-sapper treatment improved survival as a monotherapy and sensitized the tumors to checkpoint blockade.

Thus, targeting the LTβR pathway has demonstrated remarkable effectiveness in inducing TLS and improving survival in murine models, showing great promise for potential therapeutic applications in treatment of human cancer.

## Exploring the potential synergy between current cancer therapies and TLS formation

Alongside the focused approach of therapeutically targeting lymphoid neogenesis to induce TLS, it is crucial to understand the impact of existing therapies on their formation and functionality. Thus, studies have been conducted in murine cancer models involving checkpoint blockade, agonistic CD40 antibody therapy (αCD40) and corticosteroids.

As previously discussed, in their humanized patient-derived organoid-based model of CRC Küçükköse et al. showed that checkpoint blockade induced TLS and that response to α-CTLA-4 therapy was dependent on B cells, tying TLS formation to the positive effects of checkpoint blockade in CRC (19). Along a similar line, treatment with an oncolytic adenovirus encoding TNFα and IL-2 resulted in TLS formation and improved responses to checkpoint blockade in subcutaneously injected HNSCC tumors (28). Although the latter paper utilizes a subcutaneous model, which is not favorable when specifically studying characteristics of TLS, these therapy-focused findings reveal that TLS and checkpoint blockade can reciprocally affect each other and potentially synergise. As a seemingly contrasting result, TLS were not induced in GL261 or CT-2A gliomas when treated with α-PD-1 (22, 24), or in PDG-Ink4a gliomas when treated with a combination of radiotherapy and α-PD-1 (29). However, TLS were induced when CD25^+^ regulatory T cells (Treg) were depleted in combination to the treatment in PDG-Ink4a gliomas (29). This suggests that certain cell types can restrict TLS formation in specific tumor types, and could be targeted to enhance anti-tumor immunity.

Stimulating B cells with αCD40 was able to induce TLS that exhibited B cell follicular organization and GC formation in GL261 glioma-bearing mice (22). However, despite the formation of mature TLS, αCD40 did not provide a survival benefit due to the systemic induction of a CD11b^+^ regulatory B cell (Breg) population that inhibited T cell responses. Similarly, another study in the murine AOM/DSS CRC model reports the presence of TLS as well as a subset of Bregs named LARS B cells, which were found to aid in immune evasion and CRC progression (30). These findings seem contradictory to those of Küçükköse et al., who found that B cells are critical in CRC clearance (19). Interestingly, although TLS contained a large proportion of B cells, the Breg subsets were not localized within the TLS themselves (22, 30). As such, these observations underline the intricacy of interplay between TLS and other immune cells, and further suggest that targeting the differences in cellular phenotype within or outside the TLS could minimize adverse events like Breg or Treg expansion while enhancing TLS formation.

Murine models of lung inflammation and metastasis have been used as tools for functional and therapeutic research. Silina et al. utilized these models to unravel the TLS-related effects observed in lung squamous cell carcinoma patients treated with corticosteroids. The study found that patients who received combined corticosteroid/chemotherapy exhibited lower TLS density and fewer mature TLS (7). To functionally prove this concept, the authors induced TLS in the lung of naïve mice through intranasal administration of Alum/ovalbumin antigen and treated the mice with low-dose dexamethasone. The treatment indeed reduced the proportion of more mature TLS, highlighting the potential impact of corticosteroids on the TME and anti-tumor immune responses.

These studies laid a strong foundation for comprehending the impact of pre-existing therapies on TLS formation, which should be built upon in the future.

## Discussion

TLS have rapidly emerged as important players in orchestrating anti-tumor immunity due to their association with prolonged survival and response to immunotherapy in human cancer (10, 11). Despite this, the bulk of available data remains correlative and we lack a thorough understanding of how these structures form in tumors. This includes which cell types and soluble factors contribute, and what oncogenic factors in tumor cells stimulate or block TLS formation. Therefore, investigating TLS development in experimental cancer models will be instrumental for obtaining functional data and developing new therapies. Importantly, identifying models that correctly recapitulate TLS formation in specific cancer types will be critical for obtaining translational data. As such, orthotopic, immunocompetent models are necessary to draw cancer-specific observations, and should be carefully chosen to correctly mirror the pattern of TLS formation observed in human tumors. Murine tumor models reported to naturally form TLS have been reviewed in **Table 1**.

**Table 1:** Summary of major TLS components in human cancers versus treatment-naïve mouse models of the respective cancer type.

				TLS Components								
Cancer Type	System	Model	Model Type	B cells	T cells	APCs	Prolife-rating cells	GC	fDCs	PCs	HEVs	References
**Breast**	Human	NA	NA	✓	✓	✓	✓	✓	✓	✓	✓	(31–35)
	Mouse	T12 (p53^–/–^)	Genetically-induced, transplanted cell line	✓	✓	–	–	–	–	–	–	(36)
**Colorectal**	Human	NA	NA	✓	✓	✓	✓	✓	✓	✓	✓	(6, 12, 37–40)
	Mouse	AOM/ DSS	Chemically-induced	✓	✓	✓	–	–	–	–	–	(13, 41)
**Gastric**	Human	NA	NA	✓	✓	✓	✓	✓	✓	–	✓	(42–44)
	Mouse	gp130^F/F^	Genetically-induced	✓	✓	–	✓	✓	✓	–	✓	(45)
**Glioma**	Human	NA	NA	✓	✓	✓	✓	–	✓	✓	✓	(22)
	Mouse	CT-2A	Chemically-induced, transplanted cell line	✓	✓	✓	✓	–	–	–	–	(22, 24)
		GL261	Chemically-induced, transplanted cell line	✓	✓	✓	✓	–	–	–	–	(22, 24)
		mGC1	Genetically-induced, transplanted cell line	✓	✓	–	–	–	–	–	–	(24)
**Lung**	Human	NA	NA	✓	✓	✓	✓	✓	✓	✓	✓	(7, 46–48)
	Mouse	KP-F	Genetically-induced	✓	✓	✓	✓	–	✓	–	✓	(49)
**Melanoma**	Human	NA	NA	✓	✓	–	✓	✓	✓	–	✓	(8, 50, 51)
	Mouse	B16; B16-OVA (visceral/i.p.)	Chemically-induced, transplanted cell line	✓	✓	✓	–	–	–	–	✓	(14)
**Pancreatic**	Human	NA	NA	✓	✓	✓	✓	✓	✓	–	✓	(52–54)
	Mouse	KPC	Genetically-induced	✓	✓	✓	–	✓	✓	–	–	(53)
		TB32048	Genetically-induced, transplanted cell line	✓	✓	–	–	–	✓	–	–	(53)

The check symbol (✓) indicates that the cell type has been reported within TLS in that cancer type/model. The dash symbol (–) indicates that the cell type has not been investigated or found within TLS in that cancer type/model. NA: not applicable; APCs: antigen-presenting cells; GC: germinal center; fDCs: follicular dendritic cells; PCs: plasma cells; HEVs: high endothelial venules.

Genetically-engineered mouse models (GEMMs) recapitulate the oncogenic drivers and the histopathological characteristics of human tumors, and have been central in unravelling cancer-specific biomarkers and tumor cell biology (55). However, when studying the interactions between tumors and the immune system, it is also crucial to ensure that the specific aspects of the immune response being examined are accurately represented. GEMM-derived tumors are usually poorly immunogenic due to a lack of neo-antigens, which are likely central in eliciting T cell responses in human cancer (56). Thus, while GEMMs are excellent research tools, they may not always represent the best alternative to study certain aspects of immune oncology such as TLS biology. As such, model selection to study TLS formation should be preceded by investigations of how these structures present in the chosen tumor models compared to their human counterpart. As an example, genetic glioma models such as PDG-Ink4a, which is induced by RCAS-vectors encoding PDGFB in Nestin-Tv-a; *Ink4a/Arf^-/-^* transgenic mice, better recapitulate the mutational landscape of glioma patients compared to the chemically-induced GL261 glioma (29). However, TLS are absent in the untreated setting in PDG-Ink4a mice, suggesting that the immune response may differ to the one of human glioma, where TLS are present in treatment-naïve patients (29). In contrast, syngeneic orthotopic glioma models such as GL261 and CT-2A can form TLS (22, 24), and despite their high mutational burden, their immune profiles resemble the one of human GBM (57, 58). Enhancing GEMMs to more closely mimic the human anti-tumor immune response is an appealing strategy. For instance, introducing tumor antigens in autochthonous murine lung cancer using conditional GEMMs has proven effective to study endogenous T cell responses (59) and allowed for the formation of TLS (49), which are also found in lung cancer patients (7, 46–48). 

**Figure 1:**
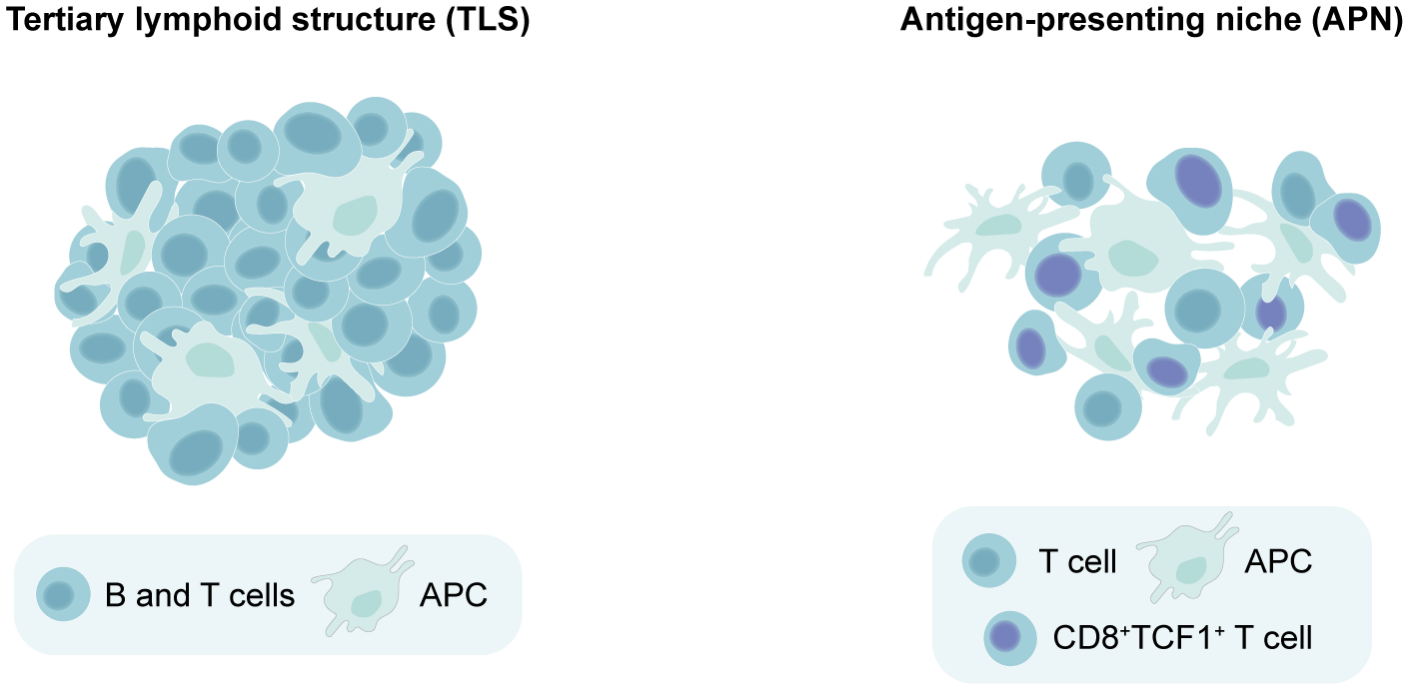
Distinguishing between tertiary lymphoid structures (TLS) versus antigen-presenting niches (APNs) based on spatial organization. Minimal requirements for identifying a lymphoid aggregate as a TLS include dense lymphocyte clustering with a defined border, containing at least T cells, B cells and antigen-presenting cells (APCs). On the other hand, APNs can be identified as immune niches that are more loosely clustered and lack a defined border, which should contain CD8^+^TCF1^+^ T cells in close proximity to APCs.

The fact that TLS maturation and composition vary across different cancer types has rendered the task of determining whether an aggregate of immune cells qualifies as a TLS more challenging. This complexity is further magnified when studying TLS in murine models, which have been less frequently utilized than patient tissues for this purpose. Additionally, it has become clear in recent years that lymphoid aggregates in cancer are not limited to TLS alone. Antigen-presenting niches (APNs) have been identified in human cancers (60, 61) and mouse models (24, 62). APNs consist of CD8^+^TCF1^+^ stem-like T cells and APCs which co-localize within the tumor and exhibit a less-organized and loose structure compared to the compact and well-organized TLS (24, 60). In line with this, quantification of TLS is typically accomplished by counting the number of these structures (6, 22, 24), as they can be easily distinguished from their surroundings. On the other hand, APNs are often assessed by measuring the proximity between APCs and CD8^+^TCF1^+^ T cells (24, 60, 62). Due to some similarities between these aggregates, misclassification across the two categories can occur as the field continues to gain insight into their characteristics. For instance, preclinical studies using subcutaneous B16 or B16F-10 melanoma models describe the induction of TLS following therapies aimed to improve DC function, including artificial adjuvant vector cells (aAVCs) (63), STING adjuvants (64) and oncolytic adenoviruses carrying IL-15 (Ad-IL15) (65). These studies identified TLS by staining for T cells, dendritic cells (DCs) and HEVs without incorporating a B cell marker, and two of them classified the induced structures as “non-classical TLS” (64, 65). However, their composition and loosely clustered appearance suggests a closer resemblance to APNs. Similarly, in murine PyMT and E0771 breast cancer models, Hua et al. found that they could boost CD8^+^TCF1^+^ immune niches forming around TA-HEVs using immunotherapies, but designated these structures as tertiary lymphoid-like structures (TLLSs) rather than APNs (66). As such, clearer guidelines to differentiate TLS from other lymphoid aggregates are needed, starting from their level of clustering and organization. We propose that TLS can be defined as densely clustered immune aggregates with a defined border that can be present either intratumorally or peritumorally, and contain at least T cells, B cells and APCs (**Figure 1**). The maturity of TLS can be further judged by the presence of HEVs, fDCs and GCs. On the other hand, APNs can be identified as intratumoral immune niches that are more loosely clustered and lack a defined border, containing CD8^+^TCF1^+^ T cells in close proximity to APCs (**Figure 1**).

**Figure 2:**
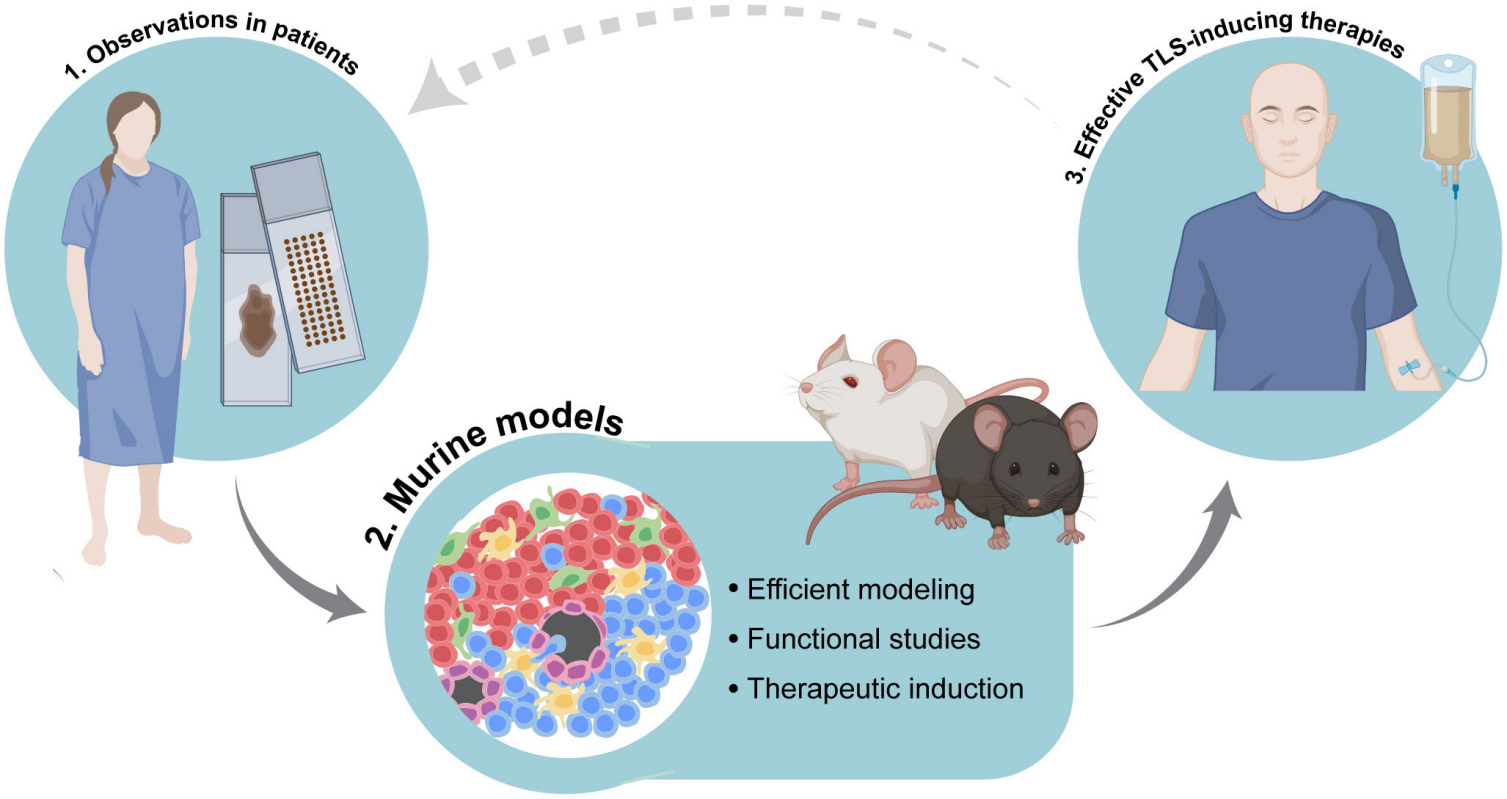
Integration of accurate murine modeling of tertiary lymphoid structures (TLS) in the cancer setting to pave the way for effective TLS-inducing therapies. Combination of observational studies of TLS in tissues obtained from cancer patients with functional studies in dynamic experimental systems will help to elucidate the mechanisms of tumor-associated TLS formation as well as the precise biological role and function of TLS in anti-cancer immune responses. This integrated approach will pave the way for the development of therapies that can successfully induce TLS and boost anti-tumor immunity. Image partially made using BioRender icons.

To summarize, TLS are complex, heterogenous structures that include multiple cell types, and whose functional investigation requires dynamic systems that can be manipulated. Combining observations of TLS in human tumors with their modeling in the murine context will be instrumental to tease apart the mechanisms regulating their function and formation. This integrated approach will build the pillars to the development of therapies that can successfully boost anti-tumor immunity (**Figure 2**). Paramount to this aim will be the judicious selection of pertinent murine models, coupled with the adherence to clear guidelines to discriminate different types of immune niches.

## Author contributions

AV: Conceptualization, Visualization, Writing – original draft, Writing – review & editing. Tv: Conceptualization, Visualization, Writing – original draft, Writing – review & editing. MR: Writing – original draft, Writing – review & editing. ME: Writing – review & editing. AD: Conceptualization, Funding acquisition, Supervision, Writing – original draft, Writing – review & editing.
